# Issue Information

**DOI:** 10.1002/hsr2.71

**Published:** 2019-10-24

## Abstract

No abstract is available for this article.

